# Conversion surgery for metastatic gastric cancer at 2 years after initial diagnosis of cancer of unknown primary with metastasis of cervical lymph nodes and ovary: a case report

**DOI:** 10.1186/s40792-021-01145-3

**Published:** 2021-03-04

**Authors:** Masaki Suzuki, Hisashi Hosaka, Yasuyuki Fukai, Yasushi Mochida, Daigo Ozawa, Norimichi Kogure, Kazunosuke Yamada, Hitoshi Ojima

**Affiliations:** 1 Department of Gastroenterological Surgery, Gunma Prefectural Cancer Center, 617-1, Nishimach, Oota, Gunma 373-0828 Japan; 2 Department of Gastroenterology, Gunma Prefectural Cancer Center, Oota, Gunma Japan

**Keywords:** Gastric cancer, Cancer of unknown primary, Conversion surgery, Distal gastrectomy, Cervical lymph node metastases, Ovarian metastases, Krukenberg tumor

## Abstract

**Background:**

Patients with stage IV gastric cancer have a poor prognosis despite improvements in intensive treatment regimens, including chemotherapy. Recently, conversion surgery has received much attention as it can provide long-term survival in stage IV gastric cancer patients who are responsive to chemotherapy. Herein, we describe the case of a patient who underwent conversion surgery for metastatic gastric cancer that was performed over 2 years after an initial diagnosis of cancer of unknown primary (CUP) with metastasis of the cervical lymph nodes and the ovary.

**Case presentation:**

A 67-year-old woman with cervical lymphadenopathy was referred to our hospital. Computed tomography showed left cervical lymphadenopathy and bilateral ovarian enlargement. Endoscopic survey revealed no signs of malignancy in the upper or the lower gastrointestinal tract. Pathological findings after cervical lymphadenectomy revealed a signet-ring cell carcinoma and were suggestive of gastric cancer metastases. However, multiple evaluations yielded no evidence of gastric cancer and the patient was diagnosed with CUP. She was prescribed chemotherapy for gastric cancer and underwent bilateral oophorectomy after undergoing chemotherapy for 18 months. Pathologic analysis of oophorectomy tissue revealed findings identical to those seen in the cervical lymph nodes. At about 2 years after the initial diagnosis, an esophagogastroduodenoscopy revealed evidence of gastric cancer. We performed a distal gastrectomy with D2 lymphadenectomy. Her postoperative course was uneventful and she remains alive with no signs of disease recurrence at 3 months post-surgery.

**Conclusions:**

To the best of our knowledge, this is the first report describing successful conversion surgery for stage IV gastric cancer in a patient whose cancer was definitively diagnosed 2 years after an initial diagnosis of CUP.

## Background

Despite early diagnosis and improved intensive treatments, gastric cancer remains a leading cause of malignancy-related death worldwide [1]. Most gastric cancer patients are not eligible for radical surgery due to the presence of locally advanced or metastatic disease [2]. With the development of multiple therapeutic approaches, standard treatment for gastric cancer is described in the Japanese treatment guidelines for gastric cancer [3]. Recently, conversion surgery has emerged as a promising strategy that can provide long-term survival in patients with stage IV gastric cancer who are responsive to chemotherapy [4].

Although there are a few case reports on conversion surgery for gastric cancer [5–7], none describe a case that was initially diagnosed as cancer of unknown primary (CUP). Here we report a case of successful conversion surgery that was performed after a cervical lymphadenectomy and a bilateral oophorectomy, wherein definitive diagnosis of gastric cancer could only be established about 2 years after an initial diagnosis of CUP.

## Case presentation

**Fig. 1:**
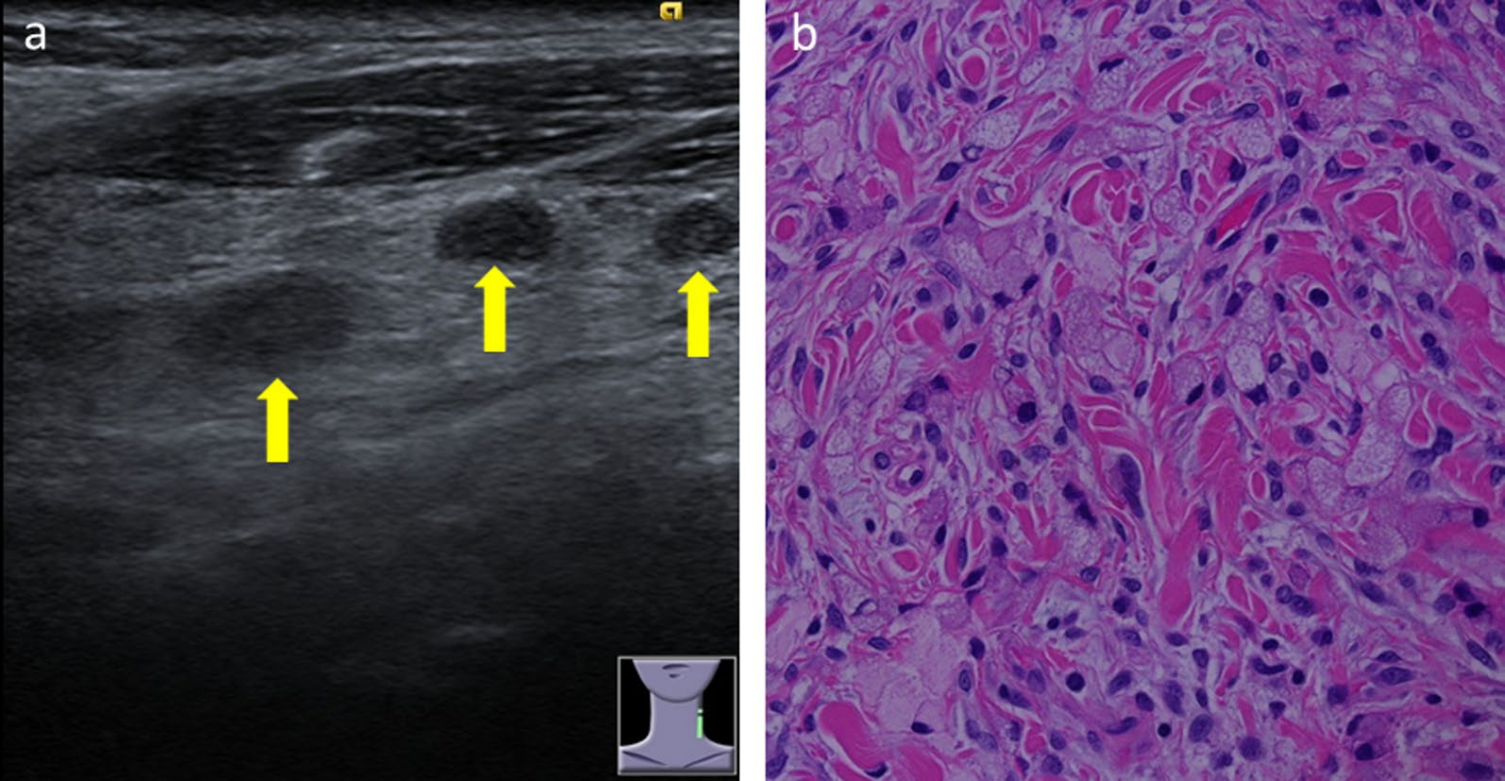
Ultrasonography and pathological findings in cervical lymph nodes. **a** Ultrasonography showed three pieces of lymph node enlargement in left cervical area (arrow). **b** Pathological findings revealed signet-ring cell and a poorly differentiated adenocarcinoma (× 400 magnification)

**Fig. 2:**
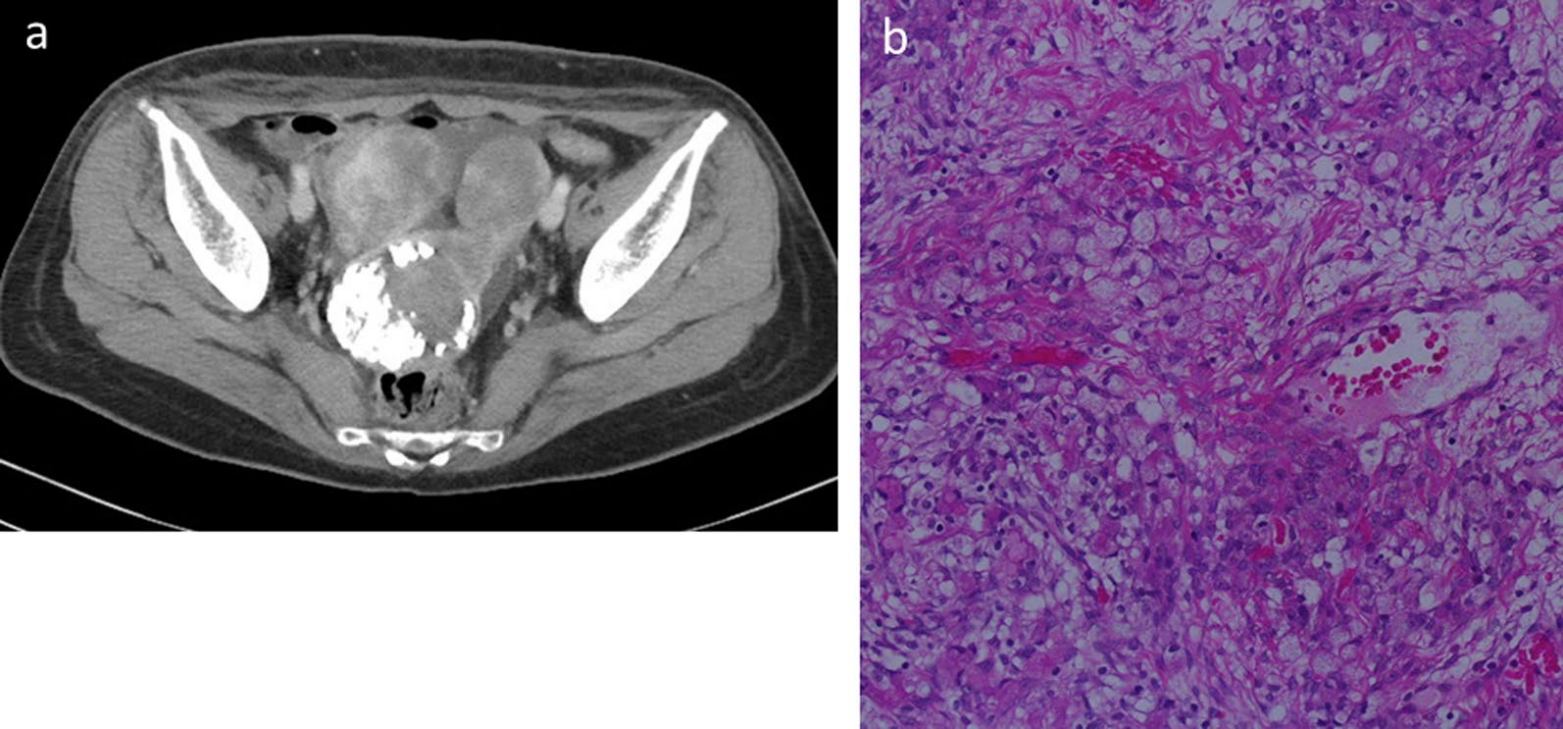
Computed tomography (CT) and pathological findings in the ovary. **a** Abdominal CT showed bilateral ovarian enlargement with uterine calcification due to myoma. **b** Pathological findings revealed signet-ring cells and a poorly differentiated adenocarcinoma (× 400 magnification)

A 67-year-old woman with left cervical lymphadenopathy was referred to our hospital for further evaluation following a diagnosis of signet-ring cell carcinoma after a needle biopsy at a local medical doctor. The patient had an Eastern Cooperative Oncology Group Performance Status of 0. Her serum carbohydrate antigen (CA) 19-9 and carcinoembryonic antigen (CEA) levels were 16.8 U/ml and 2.5 ng/ml, respectively. Ultrasonography revealed three instances of lymph node enlargement in the left cervical area (Fig. 1a). Computed tomography (CT) showed left cervical lymphadenopathy and bilateral ovarian enlargement (Fig. 2a), but without specific signs of gastric cancer or lymph node metastasis surrounding the stomach. Esophagogastroduodenoscopy (EGD) revealed only slight atrophy of the stomach and there were no findings that indicated gastric cancer. Colonoscopy was also unremarkable. To make a definite diagnosis, we performed a lymphadenectomy of the left cervical region and pathological analysis revealed the presence of signet-ring cells and a poorly differentiated adenocarcinoma, which were suggestive of metastases originating from gastric cancer (Fig. 1b). However, fluorodeoxyglucose (FDG) positron emission computed tomography (PET–CT) showed no abnormal uptake, and although we could not detect a primary lesion at that point, we recommended chemotherapy pertinent to gastric cancer.

The patient received monotherapy with oral S-1 (100 mg/body/day) for the first 4 weeks of a 6-week cycle, and after three courses of chemotherapy, CT showed a reduction in ovarian metastases without the appearance of new lesions; however, EGD continued to reveal no signs of gastric cancer. Although treatment with S-1 was effective, the patient complained of general fatigue, which was accompanied by an elevation in liver enzymes, and she was diagnosed as being allergic to S-1. Her chemotherapy regimen was switched to nab-paclitaxel (nab-PTX), which consisted of a 4-week course of intravenous nab-PTX (100 mg/body) on days 1, 8, and 15. While continuing this regimen for 18 months, the bilateral ovarian metastases remained stable. As there was no evidence of other lesions, including in the stomach, we performed a bilateral oophorectomy. There were no remarkable changes in gastric serosa and surrounding tissues of stomach and microscopic examination of the specimen confirmed a diagnosis of metastatic adenocarcinoma that consisted of a signet-ring cell carcinoma and a poorly differentiated adenocarcinoma (Fig. 2b). These findings again suggested the presence of a primary gastric lesion.

**Fig. 3:**
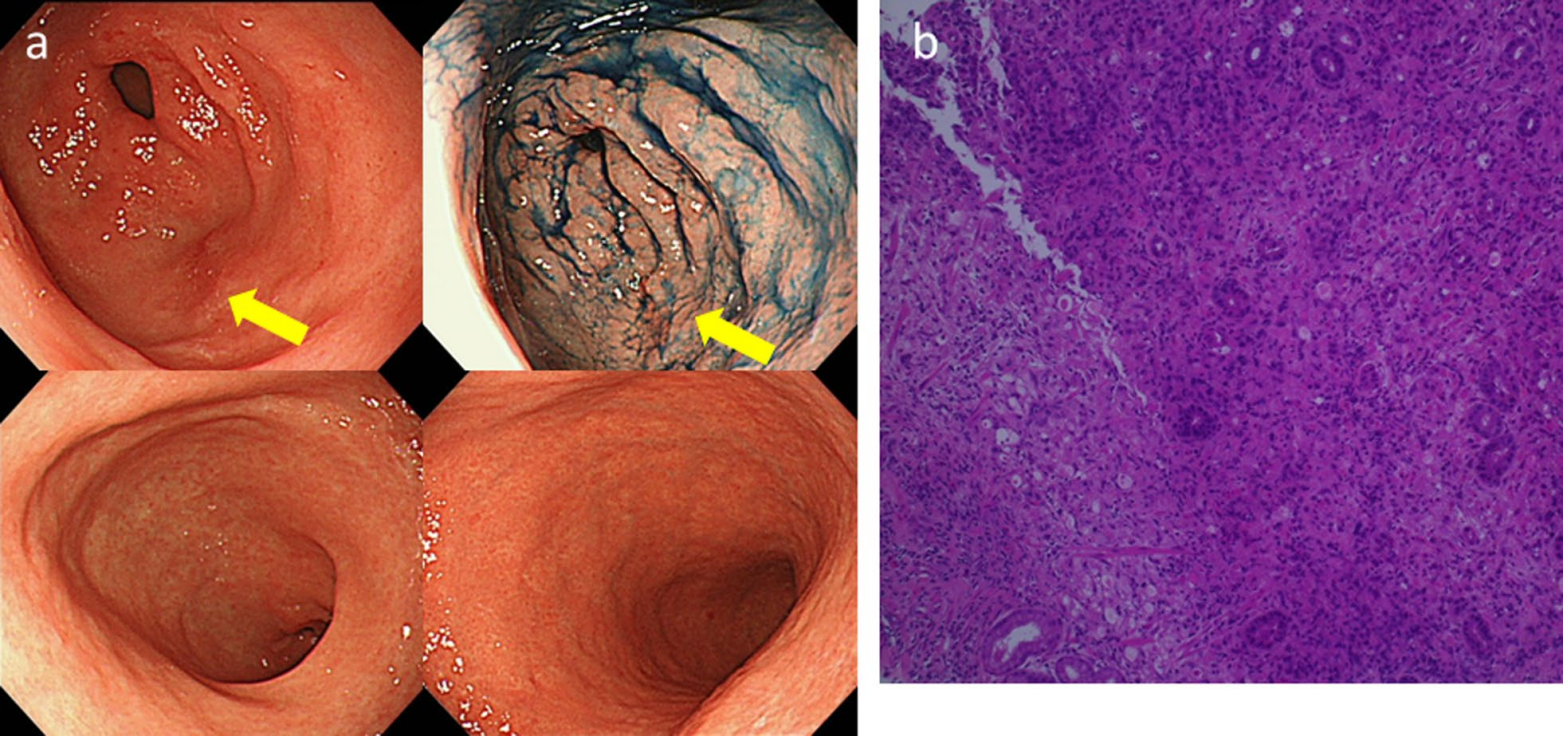
Esophagogastroduodenoscopy and pathological findings in the stomach. **a** Esophagogastroduodenoscopy showed limited rough-surfaced mucosa with slight redness changes near the pyloric ring (arrow) that stained positive for indigo carmine staining (arrow). There were no specific signs in other areas of antrum and gastric body. **b** Biopsy specimens revealed a poorly differentiated adenocarcinoma with signet-ring cells (× 200 magnification)

The patient was carefully followed up with continued chemotherapy (nab-PTX). At 3 months after the oophorectomy, we detected a limited rough-surfaced mucosa with slight redness near the pyloric ring that stained positive for indigo carmine during endoscopic examination without any abnormality in other areas of antrum and gastric body (Fig. 3a). Biopsy specimens revealed a poorly differentiated adenocarcinoma with signet-ring cells (Fig. 3b), and this was considered as evidence of gastric cancer. Nonetheless, CT showed no specific changes in the stomach or in the nearby lymph nodes and PET–CT also showed no abnormal uptake in the whole body. We discussed the possibility of a R0 resection and decided to perform conversion surgery. We discussed the method of surgery, and decided to proceed with distal gastrectomy considering the postoperative nutrition and absence of obvious signs that indicate the extent of the cancer in the upper area.

**Fig. 4:**
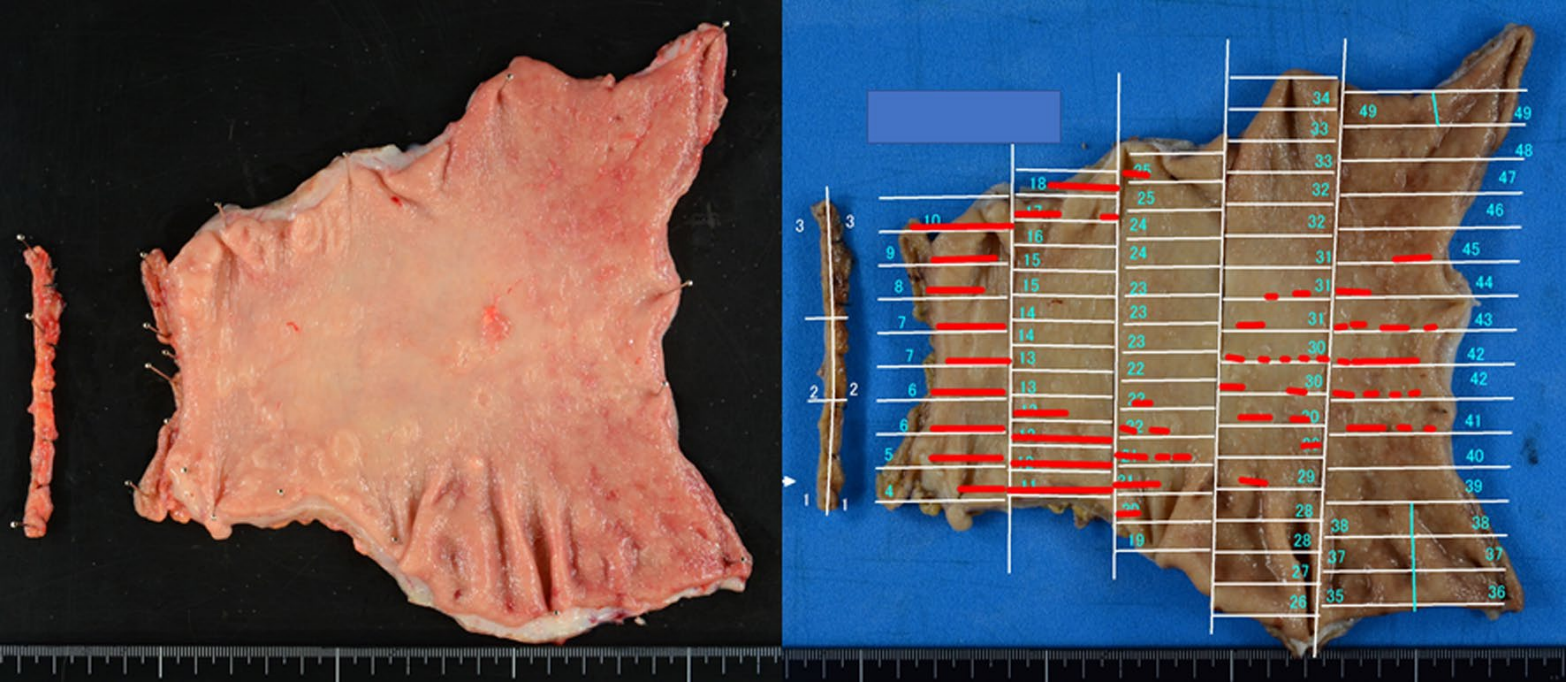
Macroscopic findings in the resected stomach. Cancer cells were seen only at the range of the red lines with no involvement of the proximal or distal margins

Thus, a definitive diagnosis could be established only about 2 years after the initial diagnosis of CUP and the patient underwent a distal gastrectomy with D2 lymphadenectomy. Detecting the cancerous area based on macroscopic findings was challenging (Fig. 4) and we did not find lymph node enlargement or peritoneal metastases intraoperatively. Pathological evaluation was graded as type 5 with T3 invasion that consisted of a poorly differentiated adenocarcinoma with signet-ring cells. The histological response of the primary and lymphatic tumor was grade 1a. Although the cancerous tissue was spread over a wide area with multiple lymph node metastases (27/32) (N3b), unexpectedly, both proximal and distal margins and the peritoneal washing were negative for cytology, according to the Japanese Classification of Gastric Carcinoma [3]. Her postoperative course was uneventful, and the patient was discharged on postoperative day 12. Currently, the patient is alive with no sign of disease recurrence at 3 months post-surgery.

## Conclusions

We describe a case of metastatic gastric cancer that could be successfully treated with chemotherapy and conversion surgery. To the best of our knowledge, this is the first report of stage IV gastric cancer being managed by conversion surgery, wherein the initial diagnosis was CUP and a definitive diagnosis could only be established 2 years later.

CUP is defined as histologically confirmed metastatic tumors whose primary site cannot be identified upon standard pretreatment evaluation [8]. CUP occurs roughly equally in men and women, with an average age-at-diagnosis of 60 years, and accounts for 3% to 5% of all tumors [8]. It has been reported that a poorly differentiated adenocarcinoma infiltrates the submucosal layer without destroying the structure of the stomach wall, which is called a type of gastric linitis plastica [9]. Macroscopically, this is characterized by a diffuse thickening of the gastric wall, and microscopically, by the existence of poorly differentiated adenocarcinoma or signet-ring cells. Specimens obtained from a standard endoscopic biopsy rarely provide confirmative diagnosis because lesions in the submucosa are difficult to reach using forceps; therefore, other reported options used to obtain samples for a definite diagnosis include endoscopic ultrasound or the endoscopic submucosal dissection technique [10, 11]. Moreover, PET–CT, which is valuable for making diagnoses in many cancers, has been reported to be a poor detector of gastric cancer, especially signet-ring cell carcinoma [12], because signet-ring cell carcinoma are rarely positive for glucose transporter 1 expression, which is related to cellular FDG uptake. In the present case, we could not initially detect any evidence of gastric cancer and methods that can identify such cancers in their early stages must be explored. Nonetheless, we suspected the possibility of gastric cancer based on pathological findings of the biopsied cervical lymph nodes and the bilateral ovarian metastases, which pointed to gastric origins of the cancer. Thus, we had appropriately prescribed gastric cancer chemotherapy in our patient.

Krukenberg tumor (KT) is a malignancy that has metastasized to the ovaries, and it is well known that gastric cancer is the leading primary lesion for KT [13]. Previous studies have shown that the prognosis in KT is poor, with median survival ranging from 9 to 11 months [14]. No optimal treatment strategies or guidelines have been established for patients diagnosed with KT of gastric origin, and even though recent studies have reported on the efficacy of oophorectomy, a survival benefit due to the procedure remains controversial [15, 16]. In our patient, as we did not detect any metastases other than those in the ovary for a long time after initiating chemotherapy, we concluded that the lesion was limited to the ovary and that we could control the disease by performing an oophorectomy.

Conversion surgery for gastric cancer has emerged as a promising therapeutic tool. It is defined as surgical treatment aimed at achieving an R0 resection after chemotherapy for tumors that were originally unresectable or only marginally resectable due to technical and/or oncological reasons [17]. In colorectal cancer patients, a surgical approach to metastatic lesions has played a crucial role in prolonging survival [18, 19]. In contrast, the definition of conversion therapy and the indications for the procedure are yet to be clarified in patients with gastric cancer. Nevertheless, Yoshida et al. have proposed a system to help clarify these indications for conversion therapy [20] wherein they divided stage IV gastric cancer patients into four categories based on the biology and the heterogeneity of the tumors. Importantly, they have specifically highlighted the impact of the existence of macroscopic peritoneal dissemination. In our case, lymphatic metastases were limited to the left cervical region and distant metastases were only the KT, i.e., no metastasis to the liver or lung. Although classically it was thought that direct seeding across the abdominal cavity accounted for the spread of KT, lymphatic dissemination has also been demonstrated [21]. In fact, our patient had no signs of peritoneal dissemination as her peritoneal washing cytology grade was CY0. Therefore, the cancer in our patient could be classified as category 2, which is defined by Yoshida et al. as the most responsive to conversion surgery [20].

Recently, treatment choices in systemic chemotherapy have increased significantly, such as molecular-targeted drugs and immune checkpoint inhibitors [22–24]. The importance of conversion surgery, which consists of a combination of chemotherapy and surgical treatment, is expected to grow in the future. Although we could not detect the primary lesion in our patient for about 2 years, careful follow-up can indicate the need for surgical resection without delay. A proper survey is indispensable in treatment regimens that aim to provide a good prognosis. We successfully used R0 surgery in our patient, which, according to the Japanese Classification of Gastric Carcinoma, is defined by the presence or absence of residual tumor after surgery [3]. However, poorly differentiated adenocarcinoma is known to have a poor prognosis [25] and our patient had multiple lymph node metastases. Therefore, careful and continuous follow-up is required.

In summary, we describe the case of a patient who underwent conversion surgery for stage IV metastatic gastric cancer, which could only be definitively diagnosed about 2 years after an initial diagnosis of CUP with metastasis of the cervical lymph nodes and the ovary. We suggest that conversion surgery, while challenging, is a promising treatment strategy for stage IV gastric cancer.

## Data Availability

The data supporting the conclusions of this article are included within the article.

## Abbreviations

CUP: Cancer of unknown primary
CT: Computed tomography
EGD: Esophagogastroduodenoscopy
FDG: Fluorodeoxyglucose
PET–CT: Positron emission computed tomography
nab-PTX: Nab-paclitaxel
KT: Krukenberg tumor
